# Diseases of the Summer and Autumn of 1845

**Published:** 1845-11

**Authors:** 


ILLINOIS

MEDICAL & SURGICAL JOURNAL.

VOL. II. NOVEMBER, 1845. NO. 8.

### DISEASES OF THE SUMMER AND AUTUMN OF 1845.

The past season has been marked, throughout the whole of Northern Illinois, and, as far as we are informed, in the adjoining States and Territories, by an unusual prevalence of disease, unequalled since the well known "sickly season" of 1838. The diseases mostly prevalent, have been Intermittent and Remittent Fevers, which, however, have not by any means been so fatal as those of the period just mentioned.

The Summer was unusually warm, but the drought, felt in many parts of the country so severely, did not extend to this region. But few cases of these diseases were observed before the month of July, during which, a very considerable number of cases occurred; the attacks increasing in frequency and severity during the month of August, and gradually diminishing, so as almost to cease in September. As usual, gastric and intestinal derangements were prevalent for several weeks before the appearance of these fevers, varying, however, in intensity, from those of the mildest character to the most severe cases of Cholera Morbus, Diarrhœa, and Dysentery. These were also the uniform forerunners of the febrile diseases, prevalent in former years. In regard to the Intermittents of the past summer and autumn, we have noticed nothing peculiar; they have been mostly of the mildest character, and arrested almost universally by the sul. quinine, in the dose of from ten to fifteen grains, administered immediately before the accession of the paroxysm. A repetition of the dose was but rarely required. An attack of the Remittent fever, was usually preceded by diminution or loss of appetite, with diarrhœa, alternating with costiveness. These, we have ascertained in numerous instances, to be the first perceptible deviations from a state of health, and they were succeeded by the langour, lassitude, and inability to mental or muscular exertion, sometimes noticed as the first symptoms of the disease. When these had continued for a time, the patient was attacked with shivering, sometimes slight, and alternating with flushes of heat, at other times persisting and attended with trembling. Pain in the head, back, and limbs, restlessness, thirst, and nausea, were the attendants upon this stage. The hot stage soon succeeded to the cold, and in this, the symptoms we have just mentioned were increased together with the re-action and activity of the circulation. No definite limits can be assigned for the continuance of the hot stage, this varying from a few hours to two or three days, when it was followed, in most cases, by a very perfect remission, and in many, by abundant and general perspiration. The second accession of the febrile paroxysm (when it occurred) and its course, were similar to those of the first, but in nearly every case, such return was prevented by proper treatment. No cases occurred within our knowledge of a congestive form, or in which the re-action did not take place. On the whole, we should say that the Remittent fever of this season, was distinguished from those of 1838 and '39 by its greater mildness, and by the more constant presence of symptoms of gastric affection. The treatment which we found most successful, was the administration in the earliest stage of free evacuants, of Emetico-Cathartics, or a purge of Calomel without the emetic. These were followed at the earliest symptoms of remission by the sul. quinine, in quantities of from ℈j to ℈jss, given usually in doses of gr. v., repeated every three hours. This rarely failed to arrest the disease, and when it did not, had the uniform effect of greatly moderating the second paroxysm. This manner of administering the quinine was preferred after numerous trials, to the use either of smaller or larger doses, as being most effectual and attended with fewest inconveniences. In some cases of great irritability, it was combined with opium with advantage, gr. j. being added to each dose, and in others of imperfect remission with diaphoretus. We are well aware that quinine, either alone or combined with opium, has been administered in many parts of the country, in much larger doses than those we have named, but we think these sufficient and preferable for the management of the disease as it occurs in this latitude. Scarcely any cases were accompanied with such local affection as to require the use of blood-letting; and out of a great number of cases, no opportunity presented itself of studying the morbid anatomy of the disease. This diminished mortality from that of former years, we ascribe principally to two sets of causes: 1st. To the use of a more judicious practice, instead of that by drastic purgatives, which formerly prevailed. 2d. To the milder character of the disease, induced by the more general cultivation of the country, and the amelioration of the condition of the inhabitants. We have abstained from a more detailed description of the disease, from its being so familiar to our readers, and from all speculations upon its cause which are of little practicable utility. We may add, however, that the Intermittent and Remittent fevers, from the manner in which they appear in common, as well as from the effects of similar treatment in both, are to be regarded only as different forms of one and the same affection. D. B.

